# Role of Notch Signaling Pathway in Glioblastoma Pathogenesis

**DOI:** 10.3390/cancers11030292

**Published:** 2019-03-01

**Authors:** Riccardo Bazzoni, Angela Bentivegna

**Affiliations:** 1 Stem Cell Research Laboratory, Section of Hematology, Department of Medicine, University of Verona, Pz.le Scuro 10, 37134 Verona, Italy; riccardo.bazzoni@univr.it; 2 Program in Clinical and Experimental Biomedical Sciences, University of Verona, 37134 Verona, Italy; 3 NeuroMi, Milan Center for Neuroscience, Department of Neurology and Neuroscience, San Gerardo Hospital, University of Milano-Bicocca, 20900 Monza, Italy; 4 School of Medicine and Surgery, University of Milano-Bicocca, via Cadore 48, 20900 Monza, Italy

**Keywords:** glioblastoma, GSCs, Notch signaling pathway, new therapeutic approaches

## Abstract

Notch signaling is an evolutionarily conserved pathway that regulates important biological processes, such as cell proliferation, apoptosis, migration, self-renewal, and differentiation. In mammals, Notch signaling is composed of four receptors (Notch1–4) and five ligands (Dll1-3–4, Jagged1–2) that mainly contribute to the development and maintenance of the central nervous system (CNS). Neural stem cells (NSCs) are the starting point for neurogenesis and other neurological functions, representing an essential aspect for the homeostasis of the CNS. Therefore, genetic and functional alterations to NSCs can lead to the development of brain tumors, including glioblastoma. Glioblastoma remains an incurable disease, and the reason for the failure of current therapies and tumor relapse is the presence of a small subpopulation of tumor cells known as glioma stem cells (GSCs), characterized by their stem cell-like properties and aggressive phenotype. Growing evidence reveals that Notch signaling is highly active in GSCs, where it suppresses differentiation and maintains stem-like properties, contributing to Glioblastoma tumorigenesis and conventional-treatment resistance. In this review, we try to give a comprehensive view of the contribution of Notch signaling to Glioblastoma and its possible implication as a target for new therapeutic approaches.

## 1. Introduction

Glioblastoma is the most common and fatal type of primary brain tumor [1]. It comprises 70% of all gliomas and is classified as a Grade IV astrocytoma (World Health Organization classification) [2]. The current standard of care for Glioblastoma patients includes maximal safe resection, followed by concurrent radiotherapy (RT) and chemotherapy with temozolomide (TMZ), followed by adjuvant TMZ [3]. Despite these aggressive therapies, the median survival time is currently 14.6 months [4], with a final mortality rate of close to 100% [5]. The failure of current therapies is due to the coexistence of heterogeneous tumor cell populations with different grades of differentiation [6,7] and the presence of a small subset of tumor cells that display stem cell-like properties, which are responsible for tumor relapse after conventional treatments [8,9]. This population, known as glioma stem cells (GSCs), exhibits an enhanced self-renewal capacity, compromised differentiation, and in vivo tumorigenicity, besides being radio- and chemoresistant [10,11]. Recent studies have demonstrated that Notch signaling is highly active in GSCs, contributing to suppressing differentiation and maintaining stem cell-like properties. Therefore, this review aims to discuss the role of Notch signaling in Glioblastoma pathogenesis and in the development of potential therapeutic strategies.

## 2. Notch Signaling Pathway Overview

**Figure 1:**
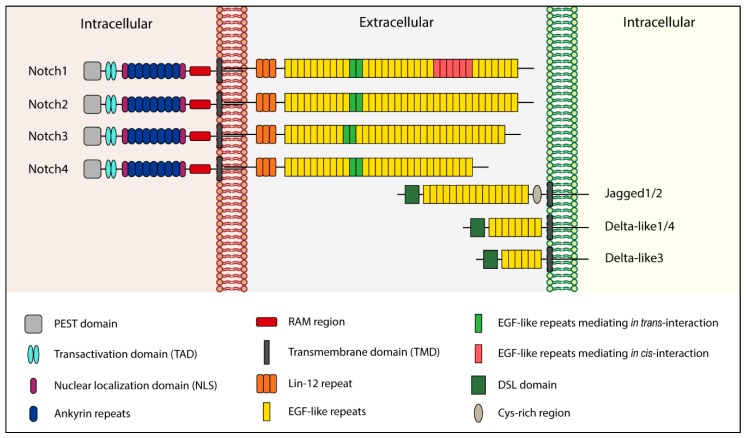
Structure of human Notch receptors and ligands.

Notch signaling is an evolutionarily conserved pathway that plays a critical role in various cellular and developmental processes, including cell proliferation, apoptosis, stem cell maintenance, cell fate decision, and tissue homeostasis [12,13,14]. Notch functions as a cytoplasmic receptor and, in mammals, there are four homologous proteins known as Notch1, Notch2, Notch3, and Notch4, which can bind two ligands families: Delta-like (Dll1-3 and -4) and Jagged (Jagged1 and -2) [15]. Both the receptors and ligands are single-pass transmembrane proteins (Figure 1). The interaction between Notch and its ligands can occur in two ways: In trans, when they are present on neighboring cells, or in cis, when the receptor and ligand are present on the same cell [16,17]. In the first case, binding leads to pathway activation, while in cis form interaction inhibits the signaling cascade [18]. 

Structurally, the Delta and Jagged ligands are very similar: They lack an intracellular domain, while their extracellular portion is constituted by 6–16 EGF-like repeats [19] and a distal Cys-rich region called the Delta/Serrate/Lag-2 (DSL) domain [20]. The DSL region is responsible for interaction with Notch receptors [21]. Moreover, Jagged1 and -2 possess a Cys-rich region closer to the membrane [21]. Unlike Delta and Jagged, the extracellular domain of Notch receptors is organized in 29–36 EGF-like repeats [19,21] and exists in a Cys-rich region called Lin-12 that noncovalently binds the extracellular Notch with the membrane-tethered intracellular Notch [18]. The Notch intracellular domain (NICD) is composed of an RBPJK associate molecule (RAM) region, repeated structural motifs named ankyrin repeats (which mediate the interaction between NICD and CBF1, a transcriptional activator), a transactivation domain (TAD), and a PEST domain (involved in Notch degradation) [21]. 

**Figure 2:**
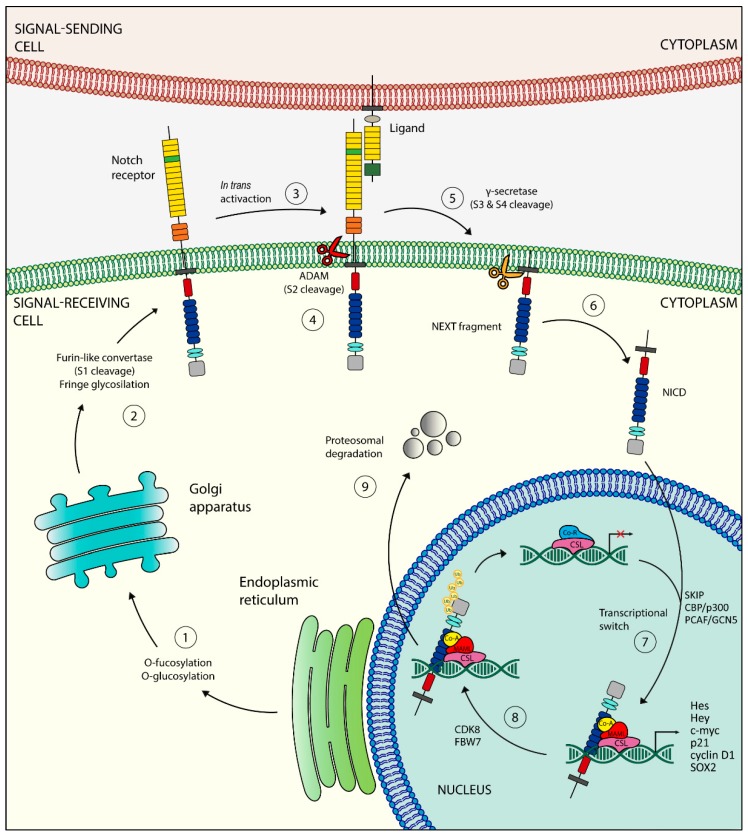
Schematic representation of the Notch signaling pathway. Once synthesized in the endoplasmic reticulum (①), the inactive single peptide precursor moves to the Golgi where it is cleaved by a furin-like convertase (S1 cleavage) (②) and translocates into the cell membrane. The binding with a Notch ligand (③) induces the second cleavage (S2) by a member of the disintegrin and metalloproteinases (ADAM) family (④), resulting in the formation of a membrane-tethered Notch truncated (NEXT) fragment, which is further processed in two sites (S3 and S4) by a presenilin-dependent γ-secretase complex (⑤), generating the Notch intracellular domain (NICD), the active form of the Notch receptor (⑥). The NICD can now enter into the nucleus, where it exerts its transcriptional activity (⑦). The ubiquitination of the NICD (⑧) leads to its proteasome degradation (⑨).

The life of Notch receptors begins in the endoplasmic reticulum (ER), where they are synthesized as inactive single-peptide precursors (Figure 2). 

Once in the trans-Golgi, a furin-like convertase proteolytically cleaves the inactive precursor [22]. This first cleavage site (S1) allows the mature Notch receptor to form, which is able to translocate in the cytoplasmic membrane and interact with its ligands [22]. The in-trans activation triggers a conformational change in the Notch receptor that exposes the second cleavage site (S2) to a member of the disintegrin and metalloproteinases (ADAM) family (ADAM10 or ADAM17) [23,24]. The result is a membrane-tethered Notch-truncated (NEXT) fragment [24], which is further processed into two sites (S3 and S4) by a presenilin-dependent γ-secretase complex, constituted by presenilin 1 (PSEN1) or PSEN2, nicastrin, presenilin enhancer 2 (PEN2), and anterior pharynx-defective 1 (APH1) [25,26]. These cleavages release the active form of the NICD into the cytoplasm. The NICD can then translocate into the nucleus [27], where it binds several DNA-binding proteins of the CSL family (RBPJK/CBF1/KBPF2 in mammals). The newly formed complex switches from a transcriptional-repressor state to a transcriptional-activator one, recruiting the transcriptional coactivator mastermind-like protein (MAML), the Ski-interacting Protein (SKIP), and histone acetyltransferases CBP/p300 and PCAF/GCN5 [28,29,30]. All these events allow the transcription of transcriptional repressors such as the Hairy Enhancer of Split (Hes) family of proteins and HES-related proteins (Hey) [31,32], two families responsible for lineage-commitment decisions. Other Notch target genes include p21/Waf1, cyclin D1 and -3, c-Myc, HER2, Notch-regulated ankyrin repeat protein (NRAR), NF-κB, pre-Tα, IGF1-R, survivin, Snail homolog 2 (SLUG), SOX2, and PAX5 [33,34,35,36]. The Notch-mediated transcriptional activation ends with the degradation of the NICD. The mechanism consists of the phosphorylation of a degron within the PEST domain of NICD, mediated by cyclin-dependent kinase 8 (CDK8) and targeted for proteasome-mediated degradation by E3 ubiquitin ligases SEL10 (also known as FBW7) [18,37]. 

In addition to canonical signaling activation, several groups have identified a novel way to activate the Notch pathway through a group of different, unrelated proteins that lack the DSL domain [38,39]. These proteins can be membrane-integral (for example, the Delta/Notch-like epidermal growth factor-related receptor—DNER), a glycosylphosphatidylinositol (GPI)-linked membrane (for example NB3/Contactin6), or secreted proteins (for example, MAGP1 and -2) [40]. Notably, Notch can non-canonically exert its biological functions either in a ligand-dependent or -independent way [41]. 

## 3. Notch Signaling in Brain Development

The Notch signaling pathway plays a fundamental role in central nervous system (CNS) development, from embryonic stages to the adult brain [41]. Although Notch signaling members show differential expression patterns throughout the brain (Table 1), they are master regulators of neurogenic niches—specialized microenvironments that are able to modulate the properties of stem cells, such as their cell number, self-renewal, and fate decision, in order to avoid the depletion of the NSC pool [42,43]. Being the starting point for neurogenesis, NSCs are extremely important [44]. In the adult brain, there are two major sites for neurogenesis: The subventricular zone (SVZ) of the lateral ventricle [43,45] and the dentate gyrus (DG) in the hippocampus [46]. 

It was demonstrated that low Notch levels induce the proliferation of NSCs, followed by an exit from the cell cycle and differentiation into neurons [60]. On the contrary, high Notch levels lead to growth arrest, or even induce a quiescent state [61,62]. The cell-fate regulation of quiescent NSCs results under RBPJK activity, probably through Notch2 and Notch3, but not Notch1 [63,64]. Instead, Notch1 seems to be crucial for the active proliferation of the NSC pool, which is selectively lost during aging [43,46]. Additionally, the ablation or loss of RBPJK function shows progenitor-cell depletion in the postnatal SVZ and DG, and reduced neurogenesis [46,65]. Thus, it is plausible to conclude that Notch signaling is instrumental for the regenerative capacity of a mature brain. Concerning neuronal migration, in the cerebral cortex, reelin-DAB1 signaling avoids NICD degradation, promoting Notch signaling in order to alter neuron morphology and, therefore, perturbing their migration [66]. This interaction between reelin-DAB1 and NICD was also reported in a study by Sibbe et al., where they showed that this mechanism is essential for the correct development of the radial glial scaffold [67]. 

To explicate their functions, neurons require glial cells which surround and insulate them, providing physical support. Like neurons, glial cells differentiate from NSCs [68]. During neurogenesis, Notch receptors are ubiquitously expressed, whereas Delta ligands appear transiently on the surface of newly differentiating neurons. NSCs, when exposed to a Delta signal, tend to resist prevailing neurogenic signals, activate Notch signaling and ultimately differentiate into glial cells. Therefore, the resistance to neurogenic signals is dependent on the activation of Notch by Delta [69,70]. Recently, several studies have suggested a new model in which Notch signaling prevents all types of NSC differentiation, rather than inhibiting neural differentiation. According to this view, NSCs will choose their fate according to whichever instructive factors prevail when they are released from the influence of Delta [71]. Indeed, Notch signaling seems to have an instructive role in gliogenesis, promoting the differentiation of many glial subtypes, with the exception of oligodendrocytes. The results of in vivo studies are consistent with the notion that Notch signaling plays an instructive role in gliogenesis through their conventional basic helix-loop-helix (bHLH) targets [72,73]. Moreover, if Notch activation promotes glial differentiation, it would be expected that some Notch receptors downstream elements (for example transcriptional targets [74]) would also influence gliogenesis.

Finally, although the role of Notch signaling in brain function is complicated and controversial, all this evidence points out the essential role of Notch in several aspects of the CNS.

## 4. Notch Pathway Deregulation in Brain Tumors and Brain CSCs

Deregulation of the Notch pathway has been associated with the development of a wide range of diseases through both germline and somatic mutations. Concerning the latter events, Notch signaling mutations lead to cancer and malignancy, of which the best-known example is T-cell acute lymphoblastic leukemia [75,76]. Over the years, aberrant Notch signaling was also found in several solid tumors, including brain tumors, although its components are rarely mutated [76,77]. 

Brain tumors are a heterogeneous group of neoplasms. Among them, the most common are gliomas and medulloblastomas [2]. Several studies have reported the abnormal expression of various Notch components in brain tumors. For instance, a higher expression of ASCL1, Dll1, Notch1, -3, -4, and Hey1 correlates with a higher glioma grade and a worse prognosis [78,79,80,81], indicating that more activated Notch signaling promotes a more undifferentiated and aggressive tumor phenotype. 

In the last twenty years, the cancer stem cell (CSC) theory has said that CSCs are responsible for tumor development and growth, as well as being the reason for cancer metastasis and relapse [33,82,83,84,85]. Brain CSCs (bCSCs), or brain-tumor initiating cells (bTICs), share similarities with NSCs [86]: They are able to grow as floating aggregates (called neurospheres) in a serum-free medium [87,88], express stem cell markers (such as nestin, GFAP, β-III tubulin, and CD133) [87,89], and can differentiate into all three neural lineages [87,89,90]. Since Notch signaling is a key regulator of the NSC state, it is easy to suppose its involvement in the maintenance of the NSC tumor counterpart. For instance, intracellular modulators such as Numb4 and Numb4Δ7 regulate the expression of stem cell markers in bCSCs, despite functioning like an inhibitor and activator for Notch signaling, respectively. This assumes that Notch mediators can alter bCSC differentiation independently from Notch inhibition itself [91]. Furthermore, NICD-overexpressing bCSCs induce tumor formation in nude mice [92].

bCSCs are generally more resistant to treatment under hypoxic conditions. In support of this, the depletion of HIF-1α alters the proliferation of glioma-derived bCSCs through blocking the interaction of HIF-1α and NICD [93]. This interaction was confirmed by another group, which showed that HIF-1α displaced HIF-2α (a Notch inhibitor) from the NICD under hypoxic conditions [94]. From these data, it emerges that active Notch signaling is necessary for maintaining stem cell features and the tumorigenic potential of bCSCs, and sets the stage for promising Notch-based therapies.

## 5. Notch Signaling and Glioblastoma

### 5.1. Expression Pattern of Notch Signaling in Glioblastoma

mRNA and protein levels of Notch1, Notch4, Dll1, Dll4, Jagged1, CBF1, Hey1, Hey2, and Hes1 are higher in brain tumor cells than normal brain cells, correlating with an elevated expression of VEGF and pAKT, and reduced levels of PTEN [79,95,96,97,98]. In particular, Notch1 expression is higher in the survival of >1 year patients than <1 year [99], whereas Notch1 overexpression is associated with low overall survival (OS) [100], suggesting a controversial role of Notch1 in gliomagenesis. Moreover, Notch1 is more expressed in peritumor-tissue GSCs compared to tumor-core GSCs [101]. Notch1 and Notch4 levels correlate with those of GFAP and vimentin, respectively. Notch4 expression increases with higher-grade and primary tumors [102]. Notch2 expression levels in Glioblastoma tissue correlate with stemness genes (*nestin*, *SOX2*), astrocyte fate genes (*vimentin* and *GFAP*), and anti-apoptotic proteins (BCL6 and BCL-W), but are inversely correlated with Olig2, CNP, and PLP1 (oligodendrocyte fate) and pro-apoptotic proteins (BAX and BCLAF1) [102,103]. The overexpression of Hey1, which is associated with survival and tumor grade, might be due to the impairment of Notch and E2F signaling; it was demonstrated that its overexpression in NSCs triggers neurosphere formation and contributes to Glioblastoma proliferation [95]. On the contrary, several groups reported a weak expression of Notch1, Notch2, MAML1, and p300 in Glioblastoma [102,104,105]. Intriguingly, the impairment of Notch signaling in secondary Glioblastoma, in which Hes1 expression is almost absent, is associated with the overexpression of ASCL1. On the other hand, the activation of Notch signaling in primary Glioblastoma is associated with low levels of ASCL1, suggesting that Notch inhibition via ASCL1 upregulation might be responsible for a potential progression into secondary Glioblastomas [78].

Correlation between Glioblastoma molecular subtypes and Notch expression was also demonstrated. Concerning the mesenchymal subtype (the most aggressive one), Notch-related genes are the most highly enriched in high p-STAT3 patients, suggesting a synergy between Notch and STAT3 signaling [106]. Verhaak et al. reported that Notch signaling is highly expressed in the classic subtype [107]. The expression levels of Dll3 and Hey2 are low in proneural Glioblastomas, while the expression level of Notch1 is high [81,105,107,108], although Cooper et al. reported reduced levels of Dll3 and Hey2 [108]. Always concerning the proneural subtype, the majority of Glioblastomas with the *IDH* mutation have a proneural gene expression pattern, even if only 30% of proneural Glioblastomas have the mutation [109]. Spino et al. reported that *IDH*-mutant gliomas (mostly low grade) have high and homogenous Dll3 expression, whereas approximately half of *IDH*-wild type Glioblastomas had either no expression or only scattered cells expressing Dll3. Regardless, Dll3 expression, if present in a *IDH*-wild type Glioblastoma, is generally restricted to non-mesenchymal subtypes [110]. Notably, Jungk et al. provided a link between Notch expression and tumor location in the proneural subtype. They observed that, in *IDH*-wild-type tissue near to the SVZ, the overexpression of Hes4 and Dll3 predicts inferior OS [111]. 

Finally, even the non-canonical Notch pathway plays a role in gliomagenesis. Huber et al. found that Deltex1 (DTX1) levels were higher in Glioblastoma compared to the normal brain, inducing several pathways involved in glioma aggressiveness such as RTK/PI3K/PKB and MAPK/ERK signaling, and the anti-apoptotic protein Mcl-1 [112].

### 5.2. Epigenetic Regulation of Notch Signaling in Glioblastoma

A peculiar characteristic of epigenetic alterations is their reversibility, making them a promising therapeutic target to explore in order to reset the abnormal cancer epigenome. To date, we do not know much about the epigenetic regulation of Notch signaling in Glioblastoma. Tsung et al. showed how the methylation status of the transcription factor *HEY1* contributes to Glioblastoma pathogenesis [113]. They found low levels of methylation on CpG islands within the *HEY1* promoter across Glioblastoma specimens when compared to a healthy brain, resulting in Hey1 overexpression [113]. In support of this, treatment with sodium butyrate (NaB), a histone deacetylase (HDAC) inhibitor, on 4910 and 5310 xenograft cell lines induced Glioblastoma cell apoptosis, decreased Hey1 expression, and increased DNMT1 levels. Moreover, the knockdown of *HEY1* reduced cell invasion, migration, and proliferation [113]. Sun et al. highlighted the role of the Delta/Notch-like epidermal growth factor-related receptor (DNER), which regulates cerebellar development and neurodevelopmental interactions between Purkinje cells and Bergmann glia which express Notch via a Deltex-dependent mechanism [114]. HDAC inhibition is able to activate the DNER/Deltex signaling pathway in Glioblastoma-derived neurospheres, resulting in cell differentiation and neurosphere-growth inhibition [114]. However, due to lack of sufficient evidence relating to the epigenetic regulation of the Notch signaling pathway in Glioblastoma, to date there are no epigenetic Notch biomarkers for cancer diagnosis.

### 5.3. Role of miRNAs in Notch-Dependent Gliomagenesis

**Figure 3:**
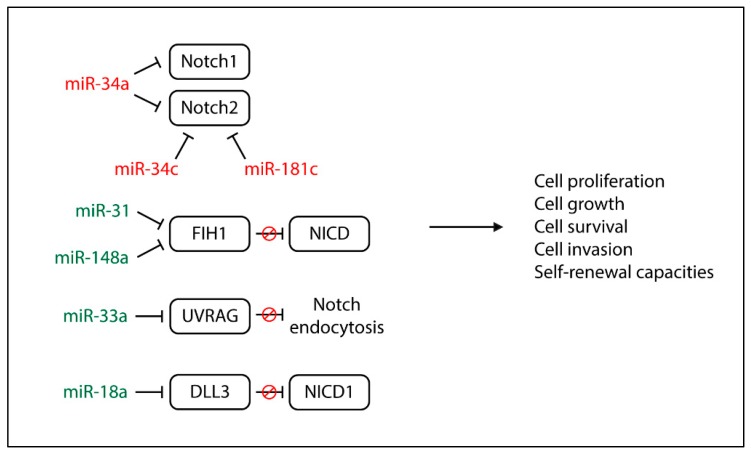
Functional effects of Notch-regulated miRNAs in glioblastoma. Red miRNAs are downregulated while the green ones are upregulated in Glioblastoma cells.

MicroRNAs (miRNAs or miRs) are small (20–22 nucleotides), non-coding RNA molecules that can play a gene-regulatory role by pairing to the mRNAs of protein-coding genes to direct the inhibition of their translation or induce their destabilization and degradation. By regulating gene expression and therefore various cell processes, like proliferation and apoptosis, their alterations are often associated with the pathogenesis of several cancers. Starting from a network topological analysis of the Glioblastoma Notch regulatory network, Sun et al. pointed out 32 miRNAs that might be involved in the Notch pathway, and six of them (miR-9, miR-34a, miR-92b, miR-124, miR-137, and miR-219-5p) might play a key role [115]. Among the Notch-related miRNAs involved in gliomagenesis (Figure 3). The miR-34 family is the most studied. It is downregulated in Glioblastoma tissue compared to normal brain tissue and is more expressed in wild-type *p53* Glioblastomas than mutant *p53* Glioblastomas [116,117].

miR-34a and miR-34a-5p function as tumor-suppressive miRNAs, inhibiting cell proliferation, cell-cycle progression, and cell invasion by targeting Notch1, Notch2, c-Met, CDK6, and EGFR [116,117]. Di Bari et al. reported that miR-34a-5p expression levels are inversely correlated to Notch1 and Notch2 expression, and its function is restored by the activation of M2 acetylcholine muscarinic receptors, which in turn downregulate Notch1 and consequently cell proliferation [117]. Wu et al. showed that lower levels of miR-34c-3p and miR-34c-5p correlate with a higher glioma grade. The overexpression of both miRNAs strongly inhibits glioma invasion and miR-34c-3p but not miR-34c-5p, promotes S-phase arrest, increases cell apoptosis, and reduces Notch2 expression [118]. Notch2 is a target of another tumor-suppressive miRNA, miR-181c, which reduces cell proliferation, cell invasion, and self-renewal capacities through Notch2 downregulation. Unfortunately, miR-181c is commonly downregulated in Glioblastoma, especially in the mesenchymal subtype, suggesting a potential relationship between miR-181c and the malignant behavior of Glioblastoma [119]. Among the miRNAs associated with shorter survival in Glioblastoma, Wong et al. discovered miR-148a and miR-31 [120]. miR-148a is frequently upregulated in Glioblastoma and correlated with hypoxia-induced and extracellular-matrix genes, while high levels of miR-31 are appreciated only in a small group of Glioblastomas and are associated with proliferation and immune-response genes. A common target of both miRNAs is factor-inhibiting HIF-1 (FIH1), which mediates their effects on tumor growth, counteracting HIF-1α and the NICD. In particular, HIF-1α is able to stabilize the NICD in order to expand and maintain GSCs. The inhibition of miR-148a and miR-31 in Glioblastoma mouse models prolongs animal survival, depletes the stem cell pool, suppresses tumor growth, and normalizes tumor vasculature [120]. With regard to GSC plasticity, miR-18a is a key player in controlling the switch between the self-renewing and non-self-renewing states [121]. By downregulating Dll3 and strengthening Notch1 signaling, miR-18a induces the expression of *SHH* and *GLI-1* via ERK, maintaining the self-renewal and stemness abilities of GSCs [121]. The last investigated Notch-related miRNA in Glioblastoma is miR-33a, which promotes GSC growth and is responsible for their self-renewal abilities. This occurs because, among miR-33a target genes, there is a UV-radiation resistance-associated gene (UVRAG) that negatively regulates the Notch signaling pathway through the repression of Notch endocytosis. Moreover, an inverse correlation between the expression levels of miR-33a and UVRAG exists in Glioblastoma, and patients with a higher expression of miR-33a are characterized by poor prognosis and shorter survival [122].

### 5.4. Tumorigenesis and Other Tumor Aspects Driven by Notch Pathway

**Figure 4:**
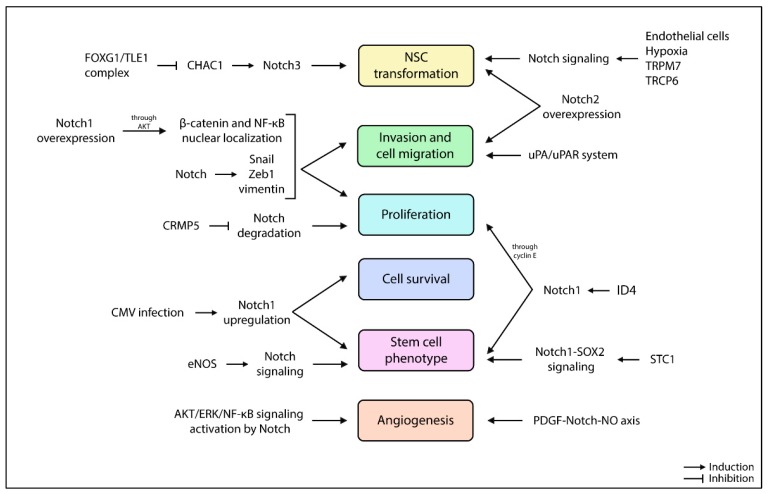
Other Notch-related tumorigenic aspects in the pathogenesis of Glioblastoma.

The overexpression of Notch1 promotes AKT activation, which in turn induces the nuclear localization of β-catenin and NF-κB, together with the Notch-mediated overexpression of Snail, Zeb1, and vimentin, promotes cell invasion and migration (Figure 4) [98]. The crosstalk between Notch and AKT is also mediated by collapsine response mediator protein-5 (CRMP5), preventing Notch degradation and favoring Glioblastoma proliferation [123]. In addition, CMRP5^high^ Glioblastoma has elevated Hey1 expression compared to CMRP5^low^ Glioblastoma, suggesting CMRP5 as an indicator of poor survival [123]. Two transient receptor cation channels, TRPM7 and TRPC6, are linked to Notch pathway-stimulating proliferation, invasion, and the migration of glioma cells [124,125]. In particular, TRPM7 levels correlate with those of Notch1, Jagged1, Hey2, and survivin [124]. Neurosphere cultures with high Notch1 levels show a more infiltrative phenotype when compared to Notch1^low^ cultures [126]; furthermore, the suppression of cell migration, tumor invasion, and angiogenesis can be achieved by targeting the urokinase-type plasminogen activator/urokinase-type plasminogen activator receptor (uPA/uPAR) system in order to inhibit Notch-signaling-induced AKT, NF-κB, and ERK pathways [127]. The oncogenic role of Notch1 can also be due to the infection of human cytomegalovirus, which upregulates Notch1, ATF5 (an anti-apoptotic protein already highly expressed in Glioblastoma), and stem cell markers CD133, nestin, SOX2, OCT4, KLF4, and BMI-1 [128]. Finally, Wang et al. reported that silencing Notch1 reduced GSC proliferation and oncogenicity in vitro and in vivo [129]. 

Besides Notch1, even Notch2 overexpression enhances cell migration in an RBPJK-dependent manner [130]. Notch2 deregulation might also promote NSC transformation and gliomagenesis, preventing neuronal lineage [103]. The stem cell phonotype is also supported and maintained by stanniocalcin-1 (STC1) and LMO2. STC1, a secretory glycoprotein, is highly expressed in glioma spheres; it is able to bind Notch1 and activate the Notch1-SOX2 signaling pathway, therefore supporting the stemness and tumorigenicity of GSCs [131]. Transcription factor LMO2, besides being inversely correlated with shorter survival, promotes the endothelial-like conversion of GSCs through the activation of VE-cadherin [132]. Jeon et al. demonstrated that inhibitor of differentiation 4 (ID4) increases Jagged1 expression, followed by Notch1 activation to drive astrocytes into a neural stem-like cell state and to increase cyclin E to produce a hyperproliferative state [133]. The downregulation of E3 ubiquitin ligase TRIM3 and the high levels of Musashi found in Glioblastoma promote the growth, survival, and self-renewal of stem cells and differentiation through the Numb/Notch pathway [134].

The tumor microenvironment also contributes to tumorigenesis. Endothelial cells function as a CSC niche by providing Notch ligands to Notch receptors on GSCs. The absence of Notch ligands on endothelial cells can reduce the CD133^+^ glioma subpopulation in vitro, inhibiting neurosphere propagation [135]. Moreover, differentiated cells within the tumor express higher levels of Dll1 compared to GSCs, contributing to Notch signaling activation in GSCs [135]. Similarly, silenced *DLL1* in GSCs decreases stem cell markers and impairs self-renewal ability [136]. On the contrary, mesenchymal stem cells (MSCs) have a tumor-suppressor effect on GSCs. Indeed, paracrine signals from MSCs sensitize NCH421k and NCH644 cell lines to TMZ, probably turning them toward more differentiated cell types, downregulating Notch1 and SOX2 and upregulating vimentin and GFAP [137]. 

Under hypoxic conditions, GSCs increases the expression of several Notch genes (*NOTCH1*, *NOTCH3*, *DLL1*, *JAGGED1*, *JAGGED2*, *HES1*, *HEY1*, and *HEY2*), and hypoxia-related genes (*HIF-1α*, *VEGF*, *LOX*, and *HIG2*) [125,138,139]. The hypoxia-Notch gene subset might hold a prognostic implication, as the overexpression of the hypoxia-Notch axis is associated with poor survival [138]. Notch1 activation under hypoxic conditions also induces the expression of transient receptor TRCP6, which has emerged as a critical player in Glioblastoma aggressiveness, promoting NFAT activity, a crucial factor for glioma proliferation [125]. Han et al. showed that Notch1 inhibition in Glioblastoma xenografts reduces the hypoxic fraction and delays tumor growth, further supporting the crosstalk between Notch signaling and hypoxia, suggesting a potential mechanism whereby Notch1 downregulation radiosensitizes Glioblastoma cells [100]. On the contrary, Bayin et al. demonstrated that the NICD is strongly expressed in perivascular tumor regions and not in the hypoxic zone. However, the authors highlighted an intratumoral GSC heterogeneity with a divergent activation of Notch signaling, which coexist in tumors, but populates distinct niches and accordingly organizes their metabolisms [140]. Charles et al. showed that endothelial nitric oxide (eNOS) maintains the GSC phenotype in perivascular niches, activating the Notch pathway via paracrine signaling and promoting in vivo tumorigenicity [141]. In another report, the convergence of the PDGF-Notch-NO axis simultaneously drove perivascular promotion of the GSC phenotype and angiogenesis [142]. Jubb et al. reported the presence of Dll4 and Jagged1 in Glioblastoma vasculature [143]. These data define Glioblastoma subsets that might be sensitive (Dll4^+^/Jagged1^+^) or resistant (Dll4^+^/Jagged1^-^) to bevacizumab, a humanized monoclonal antibody against VEGF [143]. It was also reported that the Wnt signaling pathway represses Notch signaling in a Glioblastoma hypoxic microenvironment and supports the ablation of CD133^+^ subpopulations [144], which can populate tumors regardless of local vascularity and selectively utilize anaerobic glycolysis to expand in hypoxia [140]. Moreover, Notch1-stimulated GSCs induce highly vascularized tumors in vivo, with the production of several angiogenesis-related factors and the expression of pericyte cell markers [92]. 

Finally, the FOXG1/TLE1 complex cooperates with Notch signaling to promote gliomagenesis by directly repressing CHAC1 expression, a negative regulator of Notch3. Dali et al. also identified DNER as an additional potential transcription-repression target of FOXG1/TLE1: DNER inhibits Glioblastoma-derived neurosphere growth and promotes their differentiation, opposite to the effect of FOXG1 and TLE1 [145]. 

### 5.5. Therapeutic Approaches against Notch Signaling in Glioblastoma

To date, several classes of Notch inhibitors have been developed (Table 2). The most employed in cancer are γ-secretase inhibitors (GSIs), which prevent the release of the active NICD from the receptor by the γ-secretase complex, while α-secretase inhibitors (ASIs), which inhibit members of the ADAM family by preventing the second cleavage (S2) of the Notch receptor, are less used. Finally, to overcome GSI/ASI resistance, new therapeutic approaches that directly or indirectly target Notch signaling have been developed.

#### 5.5.1. γ-Secretase Inhibitors

DAPT (GSI-IX), the most known and used GSI, amplifies the effect of radiation, and reduces GSC proliferation and the number of endothelial cells disrupting the perivascular niche [146]. The suppression of cell proliferation and induction of apoptosis is mediated by the decrease of NF-κB (p65) expression through Notch inhibition [105]. Glioblastoma neurospheres with high Notch activity are more sensitive to DAPT and DAPT-treated cultures and show a more differentiated state and low Hes5 levels [126,146,147]. The combined treatment of DAPT with Iressa, an EGFR inhibitor, reduces VEGF expression and secretion when compared to single treatments. Unfortunately, this combined treatment is not sufficient to fully block endothelial cells from sprouting, probably due to other angiogenesis factors. Moreover, by blocking EGFR signaling, Hes1 levels decreased, suggesting that EGFR signaling stimulates Notch pathway activity [148]. Like DAPT, LLNle and L-685,458 are also able to kill GSCs, inhibiting NICD generation and inducing proteasome inhibition, proteolytic stress, and mitotic arrest [149]. DAPT and L-685,458 strongly reduce neurosphere formation in Glioblastoma cell lines, although DAPT and L-685,458 work to a much lesser extent than LLNle [149,150]. GSI RO4929097, in combination with radiation and TMZ, decreases the expression of CD133, SOX2, and nestin (inducing neural and astrocytic differentiation), has an anti-proliferative effect (reducing 3D spheroid growth), and increases the survival of the orthotopic Glioblastoma mouse model [151,152]. The triple combination is more effective than radio- and chemotherapy, or GSI alone [152]. Notably, Saito et al. showed that GSCs sensitive to DAPT, RO4929097, and BMS-708163, another GSI, have a gene signature enriched in proneural genes such as *OLIG2*, *SOX2*, *ERBB3*, *HDAC2*, *TGFB3*, *DLL3*, *CHIL3I*, and *NKX2-2* [151]. 

MRK003 reduces cell growth in vitro and in vivo and sensitizes cell lines and neurospheres to radiation and TMZ [153]. It was demonstrated that MRK003 has a strong therapeutic potential in CD44^high^/CD133^low^ GICs [150]. Intriguingly, Kahlert et al., through the metabolomic analysis of MRK003-treated Glioblastoma neurospheres, found reduced levels of intracellular glutamate, glutaminase (known to promote cancer cell proliferation), phosphocoline (which is elevated in fast-dividing glioma cells and in malignant high-grade brain tumors), and glycine (involved in the survival regulation of hypoxic glioma cells) [154]. However, MRK003 and GSI-XVII reduce the expression of stemness markers, such as CD133, nestin, BMI-1, and OLIG2, concomitant with the reduction of neurosphere formation and clonogenicity ability in vitro and in vivo. Moreover, both GSIs increase apoptosis, reducing AKT and STAT3 phosphorylation [150,155], and GSCs pretreated with MRK003 and GSI-XVIII show reduced tumor formation in vivo. In order to avoid GSI gastrointestinal toxicity, Fan et al. administered inhibitors through the brain implantation of drug-impregnated polymer beads that effectively blocked tumor growth and significantly prolonged animal survival [155]. 

Finally, GSI-X significantly impairs c-CSC cell growth compared with p-CSC pools, with no effects observed in cell-cycle distribution, apoptosis, and cell-invasion assays [156].

In conclusion, GSIs, being pan-Notch inhibitors, cause intestinal toxicity through the goblet cell metaplasia of the small intestinal epithelium. To limit toxicity, a preclinical study using antibodies for specific Notch receptor has shown that inhibition of the Notch1 receptor alone induces mild goblet cell metaplasia, whereas the inhibition of Notch2 receptor alone can eliminate this effect [157]

#### 5.5.2. α-Secretase Inhibitors

In the literature, only one ASI has been used in Glioblastoma. Floyd et al. found that INCB3619 decreases cell growth and tumor size and prolongs the survival of a Glioblastoma animal model [158]. Moreover, combined treatment with DAPT represses *HES1* and *HEY1* expression, as well as *LIF* and *YKL-40* levels, two new key players in Glioblastoma pathogenesis [158,159]. 

#### 5.5.3. Other Molecules

Besides ASIs and GSIs, other molecules have been employed to block Notch signaling in Glioblastoma with the aim of overcoming side effects and resistance to GSIs.

Arsenic trioxide (ATO) is an inorganic compound that was approved by the Food and Drug Administration (FDA) in 2000 for the treatment of acute promyelocytic leukemia (APL) because of its strong anti-growth APL-derived stem cells [160]. With this rationale, Zhen et al. discovered that ATO reduces colony formation and nestin expression, induces apoptosis, and enhances the radiation-induced killing of Glioblastoma cells by decreasing Notch1 and Hes1 protein levels [161]. These data were confirmed by other studies, which further demonstrated that ATO treatment decreases CD133 expression and induces apoptosis through the repression of phosphorylation of AKT and STAT3 through the Notch pathway [162,163].

Tipifarnib, a farnesyltransferase inhibitor, sensitizes GSCs to GSIs, whereas the combined treatment with RO4929097 makes tumor cells more sensitive to radiation, resulting in significantly reduced tumor growth and improved survival in animal models. Intriguingly, non-stem GSCs are resistant to treatment, suggesting that combined treatment selectively targets the CSC pool [164].

Honokiol, a natural extract from different *Magnolia* species, can readily cross the blood-brain barrier and shows pro-apoptotic activity in Glioblastoma [165]. Combined treatment with *O*^6^-benzylguanine (O6-BG), an MGMT inhibitor, increases TMZ sensitivity and suppresses Notch3 and Hes1 mRNA levels with pro-apoptotic effects on GSCs [166].

Fibulin-3, an extracellular matrix glycoprotein, is highly expressed in Glioblastoma and functions as an autocrine/paracrine activator of NF-κB and the Notch pathway, promoting tumor invasion, angiogenesis, and drug resistance, besides being a marker of poor prognosis [167]. Nandhu et al. developed an antibody against fibulin-3 called mAb428.2, which is able to prevent the fibulin-3-activation of ADAM17, resulting in cell apoptosis, enhancement of inflammatory macrophage infiltration, reduced tumor growth and vascularization, and extended survival in Glioblastoma mouse models [167]. Always with regard to fibulin-3, Li et al. developed ZR30, an in vitro synthetized protein, based on the human fibulin-3 protein variant (ETSP), lacking the N-terminal signal peptide (responsible for extracellular exportation). ZR30 prevents MMP2 activation, thus limiting tumor invasion, blocking EGFR/Notch/AKT signaling, exerting an anti-tumor effect on different Glioblastoma cell subpopulations in a mouse model miming intratumor heterogeneity [168]. 

A dominant negative MAML peptide was developed in order to inhibit Notch signaling, and it was demonstrated that it has anti-proliferative and pro-apoptosis effects, decreasing Hes1 and Hey3 expression [153,169]. Hes and Hey family members are also downregulated by retinoic acid, which can inhibit sphere and colony formation, promote cell-growth arrest, and induce neural differentiation, both in vitro and in vivo [170].

Protein kinase C iota (PRKCi) is in close proximity with Notch1. In a study by Phillips et al., it was found that inhibiting PRKCi by aPKC-PSP results in lower Notch1 levels and increased GSC death [171].

### 5.6. Therapeutic Resistance to Notch Inhibitors

**Figure 5:**
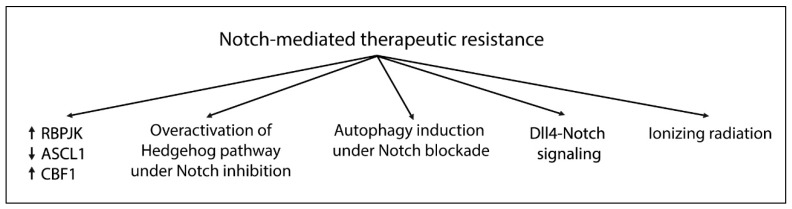
Potential mechanisms involved in Notch-mediated therapeutic resistance.

The elevated heterogeneity of Glioblastoma tissue is why the therapeutic approach fails. Concerning Notch-targeting strategies (Figure 5), it was shown that GSCs express higher levels of *RBPJK* compared to non-GSCs, and *RBPJK* knockdown reduced tumor propagation in vitro and in vivo [172]. Notably, RBPJK could regulate a different transcription program than Notch, binding CDK9 and therefore affecting Pol-II transcription elongation. Targeting CDK9, or even c-Myc, an upstream regulator of RBPJK, decreased the propagation of GSCs and prolonged survival in an orthotopic mouse model [172]. Another possible resistance mechanism is the overactivation of the Hedgehog pathway under Notch inhibition, which in turn increases GLI-1 expression via the inhibition of Hes1. Indeed, targeting both Notch and Hedgehog increases apoptosis, inhibiting cell growth and colony-forming ability more dramatically compared to monotherapy [173]. The upregulation of Dll4-Notch signaling might have a possible role in chemoresistance and be related to a classical vascular pattern, tumor edema, and MGMT-methylated promoter [97].

High CBF1 levels are also implicated in drug resistance. Maciaczyk et al. found that targeting CBF1 impairs glioma invasion, suppressing Zeb1, an activator of the epithelial-mesenchymal transition program, and sensitizes cells to drugs other than GSIs. Unfortunately, it was reported that a CBF1 blockade can activate Notch target genes [174].

It was also reported that ionizing radiation induces Notch pathway activation, resulting in GSC expansion, whereas Notch inhibition sensitizes GSCs, but not non-GSCs, to radiotherapy [175]. Similarly, CD133^high^ GSCs were resistant to MRK003 treatment due the high level of drug-resistance genes, such as *BCRP1* [176]. It was also demonstrated that numerous patient-derived GSCs, responding to Notch inhibition, express high levels of ASCL1, a proneural transcription factor involved in normal neurogenesis. ASCL1^high^ GSCs exhibit a latent capacity for terminal neuronal differentiation in response to Notch signaling inhibition, whereas ASCL1^low^ GSCs do not [177].

Finally, Nastumeda et al. found that MRK003 promotes autophagy in GSCs, as revealed by the higher expression of LC3B-II/LC3B-I autophagy markers after treatment, resulting in chemoresistance induction. Fortunately, this can be avoided by treating cells with autophagy inhibitors together with GSIs [178].

### 5.7. Glioblastoma Clinical Studies and Notch Inhibitors

In the last decade, several clinical trials have been conducted to evaluate the dose-limiting toxicity (DLT) and tumor-suppressive effects of Notch inhibitors in Glioblastoma patients. Despite different clinical studies being conducted, only two molecules were tested: RO4929097, a γ-secretase inhibitor, and CB-103, a novel first-in-class orally active small-molecule inhibitor of the Notch transcriptional activation complex in the nucleus [179].

Targeting recurrent or progressive Glioblastoma patients with GSI RO4929097 (NCT01122901; https://clinicaltrials.gov/ct2/show/NCT01122901) showed that the six-month and two-year progression-free survival was 1.7 months, while OS (two years) was 6.7 months. Moreover, according to the response assessment in the neuro-oncology (RANO) criteria, the majority of enrolled patients presented disease progression, one complete remission, and three occasions of stable disease. The lack of activity by RO4929097 is probably due to its auto-induction metabolism, resulting in an increased CYP3A4 activity and a marked reduction of steady-state drug levels [180].

Since the standard of care for glioblastoma patients is radiotherapy followed by chemotherapy, a phase 0/I was conducted to study the effect of chemo-radiotherapy in combination with RO4929097 in newly diagnosed malignant gliomas (NCT01119599; https://clinicaltrials.gov/ct2/show/NCT01119599). The combined regimen was well-tolerated and no dose-limiting toxicities were observed. The study showed a significant decrease in proliferation and NICD expression by tumor cells and blood vessels, with a concomitant reduction of the CD133^+^ GSC subpopulation. Investigators also reported alterations in the angiogenesis pathways under treatment, although the tumor was able to adapt to perturbations in the microenvironment, switching to a Notch-independent form of angiogenesis, highlighting the necessity of concomitantly targeting multiple signaling pathways in Glioblastoma [181]. Angiogenesis is the issue of another study (NCT01189240; https://clinicaltrials.gov/ct2/show/NCT01189240), in which the combined treatment of RO4929097 with bevacizumab on patients with progressive or recurrent malignant glioma was evaluated. The drug combination was well-tolerated, and two (both Glioblastoma) of 12 evaluable subjects showed a radiographic response with complete or partial remission, although bevacizumab has often been associated with an invasive phenotype [182]. 

Finally, a new clinical trial (phase I/IIA) began in January 2018 (NCT03422679; https://clinicaltrials.gov/ct2/show/record/NCT03422679). In this study, CB-103 was employed to treat several advanced or metastatic solid tumors and haematological malignancies, including Glioblastoma. The current primary outcomes are to evaluate DLT and antitumor efficacy. The study is ongoing.

## 6. Conclusions

Glioblastoma is the most aggressive types of brain tumor, with a final mortality rate close to 100%. Conventional therapies have failed to improve patient survival due to a small subpopulation of cancer cells known as GSCs that exhibit an enhanced self-renewal capacity, compromised differentiation, in vivo tumorigenicity, and resistance to radio- and chemotherapy. Blocking certain pathways can be useful to induce GSC terminal differentiation and apoptosis in order to reduce their survival. Among them, the Notch signaling pathway is one of the most important. The dysregulation of Notch signaling can take place at different levels, involving genetic, epigenetic, miRNA, and protein regulation in order to maintain cells in an undifferentiated state. Therefore, the Notch pathway could represent an attractive target for treatment in order to induce cell differentiation and kill both undifferentiated and differentiated tumor cells. The most employed and effective inhibitors in Glioblastoma are GSIs, which inhibit the activity of the γ-secretase complex, resulting in no formation of the active Notch form. Even if Notch-targeted monotherapy seems to be promising, a better accomplishment is to combine Notch inhibitors with other molecules or chemotherapeutic agents like bevacizumab and TMZ, respectively. This is because Notch signaling is at the center of a signaling network, which also includes pathways such as PI3K/AKT, NF-κB, STAT3, Hedgehog, and Wnt/β-catenin, which are involved in cell differentiation, cell growth, and survival. Although effective, GSIs present many several side effects, like acute gastrointestinal toxicity. Thus, new compounds or other administration routes should be developed to avoid side effects. To further complicate the possibility of achieving satisfactory results in patient treatment, no specific Notch-related biomarkers are available for Notch-targeted treatment selection and/or treatment response. This situation suggests that patients might be exposed to an ineffective therapeutic regimen, with consequent side effects and higher costs for health institutions.

In conclusion, the Notch pathway is definitely involved in Glioblastoma tumorigenesis, even if its role seems to be quite controversial. However, Notch-signaling inhibition represents a potential therapeutic approach to kill tumor cells, especially in combination with other molecules. Further investigations are needed to better understand the molecular mechanisms of Notch signaling in Glioblastoma pathogenesis, and how they can be overcome to develop an effective therapeutic approach against Notch to improve patient survival.

## Figures and Tables

**Table 1:** Expression pattern of Notch receptors and ligands in the adult brain.

Notch Signaling Members	Expression Pattern	References
**Notch1**	Neurons, astrocytes, precursor cells, ependymal cells, endothelium	[47,48,49,50]
**Notch2**	Precursor cells	[47,51]
**Notch3**	Precursor cells	[51]
**Notch4**	Endothelium	[52]
**Delta/Notch-like epidermal growth factor-related receptor (DNER)**	Neurons	[53]
**Dll1**	Intermediate neural progenitors, neurons	[49,51,54,55]
**Dll3**	Intermediate neural progenitors	[54]
**Dll4**	Endothelium	[56]
**Jagged1**	Precursor cells, intermediate neural progenitors, neurons	[48,49,54,57,58,59]
**Jagged2**	Neurons	[49,52]

**Table 2:** Therapeutic approaches against Notch signaling in Glioblastoma.

Class of Inhibitors	Molecules	Biological Effects							
		Decreased Cell Growth	Anti-Proliferative Activity	Pro-Apoptotic Activity	Reduced Neurosphere Formation	Reduced Colony Formation	Decreased Tumor Size In Vivo	Prolonged Animal Survival	Others
**γ-Secretase inhibitor (GSI)**	DAPT (GSI-IX)		✓	✓	✓				• Amplifies the effects of radiation • Induces differentiation • In combination with Iressa, reduces VEGF
	LLNle			✓					
	L-685,458			✓	✓				
	RO4929097		✓		✓			✓	• In combination with RT and TMZ, reduces CD133, SOX2, and nestin expression
	BMS-708163								
	MRK003		✓	✓			✓		• Sensitizes cells to TMZ • Reduces onco-metabolite levels • In combination with GSI-XVIII, reduces CD133, nestin, OLIG2, and BMI-1 expression, and neurosphere formation
	GSI-XVIII			✓			✓		
	GSI-X	✓							• No effects on cell-cycle distribution, apoptosis, and cell invasion
**α-Secretase inhibitor (ASI)**	INCB3619	✓					✓	✓	• In combination with DAPT, represses *HES1* and *HEY1* expression
**Others**	Arsenic trioxide (ATO)			✓		✓			• Enhances radiation-induced killing of Glioblastoma cells • Decreases Notch1, Hes1, nestin, and CD133 levels
	Tipifarnib			✓					• Sensitizes GSCs to GSIs • Combined treatment with RO4929097 sensitizes cells to radiation and reduces in vivo tumor growth
	Honokiol			✓					• Combined treatment with o6-BG increases TMZ sensitivity and suppresses Notch3 and Hes1 mRNA levels
	mAb428.2			✓			✓	✓	• Blocks ADAM17 activation • Enhancement of inflammatory macrophage infiltration • Reduces in vivo vascularization
	ZR30						✓	✓	• Prevents MMP2 activation • Limits tumor invasion • Blocks EGFR/Notch/AKT signaling
	dnMAML peptide		✓	✓					• Reduces Hes1 and Hey3 expression
	Retinoic acid	✓			✓	✓			• Downregulates the Hes and Hey family • Induces neural differentiation
	aPKC-PSP			✓					• Reduces Notch1 levels
